# A Superparamagnetic Platform Enhanced with Metal-Modified Carbon Dots for Rapid Dual-Modal Detection of *Staphylococcus aureus*

**DOI:** 10.3390/bios16080403

**Published:** 2026-07-24

**Authors:** Hongzhou Chen, Mengyu Li, Weichao Wu, Yang Liu, Chuanfu Lu, Qi Li, Yuwei Ren, Yingwang Ye, Baocai Xu, Kezhou Cai

**Affiliations:** School of Food Science and Engineering, Hefei University of Technology, Hefei 230009, China; hz.chen@163.com (H.C.); mengyuli01@163.com (M.L.); 15955177314@163.com (W.W.); orangeliuyang@163.com (Y.L.); lcf164140@163.com (C.L.); 18742033548@163.com (Q.L.); yuwei_ren2021@163.com (Y.R.); yeyingwang04@126.com (Y.Y.)

**Keywords:** *Staphylococcus aureus*, magnetic separation, ultra-sensitive detection, aptamer

## Abstract

*Staphylococcus aureus* (*S. aureus*) is a significant pathogen that causes foodborne diseases. Meat, with its abundant nutrients and high water activity, constitutes an ideal niche for *S. aureus* colonization. Numerous studies have shown that human digestive tract diseases caused by consuming meat products contaminated with *S. aureus* occur frequently. It is of great significance to strictly monitor *S. aureus* in meat matrices. A novel biosensor employing dual-signal output was developed through the combination of efficient magnetic separation and dual-modal precise detection. Designed for the rapid enrichment of *S. aureus*, it significantly boosts detection sensitivity and accuracy, thereby enabling the earlier identification of potential contamination sources. This method uses vancomycin-modified magnetic beads as the capture element, and aptamer-modified nanozymes as the signal element. After magnetic separation, the 3,3′,5,5′-tetramethylbenzidine color reaction can be used to quickly and sensitively detect *S. aureus*. It achieved a detection limit as low as 10 cfu/mL. Moreover, this dual-signal sensor based on efficient magnetic separation can sensitively detect *S. aureus* in meat products, thus showing good application prospects in food matrices and further improving the reliability and specificity of detection.

## 1. Introduction

*Staphylococcus aureus* (*S. aureus*) belongs to the genus *Staphylococcus* and is a typical Gram-positive bacterium. *S. aureus* is one of the main pathogenic bacteria causing common diseases in humans and animal [1,2]. *S. aureus* poses a severe threat to food safety by proliferating in food matrices and secreting enterotoxins during its metabolic processes [3,4]. These toxins enter the human body through the food chain, causing food poisoning and endangering health [5,6]. Therefore, it has long been a key focus in the supervision of food biological contaminants [7,8].

In meat matrices, the traditional detection methods (traditional cultivation methods, molecular detection methods and immunological assays, etc.) for *S. aureus* encounter numerous challenges [9,10,11]. Although traditional analytical methods are classic and reliable, their shortcomings in terms of detection speed and on-site deployment capabilities have become insufficient to meet the urgent requirements of food safety testing for immediate on-site responses. To overcome these limitations, various new identification strategies have been developed, including fluorescence method, colorimetric method, surface-enhanced Raman scattering method and electrochemical method. Yang et al. constructed an aptamer sensor based on carbonyl iron powder and multi-walled carbon nanotubes for the fluorescence detection of *Escherichia coli* O157:H7 in dairy products [12]. Kang et al. proposed that the Fe_3_O_4_@Zr-MOF/GOx nanoprobes can specifically perform colorimetric detection of *Salmonella typhimurium* [13]. Overall, nanomaterials with unique physical and chemical properties have been successfully applied in the rapid detection of foodborne pathogenic bacteria [14]. However, the performance of single-type detection principles is limited, making it difficult to achieve multiplex detection and high-sensitivity detection [15]. The single-signal detection strategy based on nanomaterials lies in relying on the signal response triggered by a single recognition. However, due to the lack of an inherent cross-validation mechanism, this mode is highly susceptible to environmental fluctuations and operator errors, resulting in a lack of reliability and reproducibility of the detection results [16]. Composite detection systems incorporating nanomaterials and biological recognition elements offer dual advantages of heightened sensitivity and multiplexed signal acquisition, which in turn allows for on-board internal calibration and scenario-responsive mode switching [17,18,19]. To overcome the limitations of single-signal readouts and achieve higher detection sensitivity and a wider dynamic linear range, Shen et al. took *S. aureus* as the target bacteria and developed a responsive ratio nanoprobe [20]. As expected, the ratio fluorescence detection of *S. aureus* by the nanoprobes showed extremely high specificity. Therefore, there is a need for accurate and reliable biosensors for the in situ detection of pathogenic bacteria in meat samples, particularly through multi-mode platforms that enable rapid and on-site analysis.

**Figure 1:**
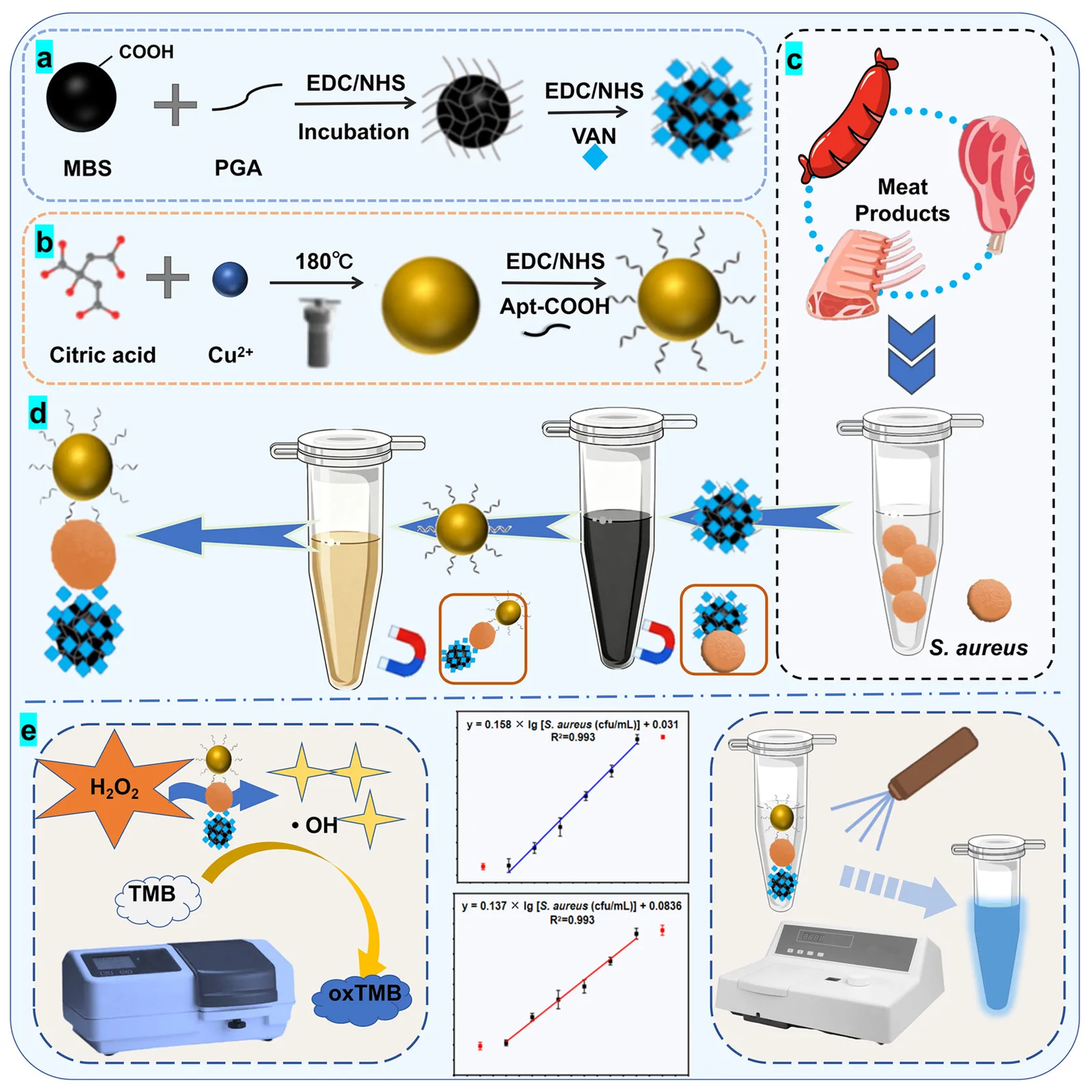
Schematic diagram of the dual-modal sensor driven by enhanced magnetic separation technology. (**a**) Synthesis schematic of VAN/PGA@MBs, (**b**) synthesis schematic of Cu@CDs/Apt, (**c**) sample pretreatment, (**d**) flowchart of dual-modal detection process, (**e**) output of dual-modal signals.

Based on the above information, we have developed a colorimetric–fluorescent dual-signal biosensor that integrates efficient magnetic separation and dual-modal precise detection. This system enabled rapid enrichment of *S. aureus* and provides a dual-signal readout, which significantly enhances both the sensitivity and accuracy of detection and facilitates earlier identification of potential contamination sources (Figure 1). We synthesized a new type of carbon dots (Cu@CDs) by doping copper elements and connected them with aptamers to achieve the identification of the analyte. This material possesses the excellent optical properties (such as highly efficient and stable fluorescence performance, which provided a basis for the dual-modal detection strategy) and biocompatibility of ordinary CDs, and also has the unique catalytic activity of metal elements, thereby achieving a synergistic enhancement in performance. In addition, we proposed a networked magnetic beads (MBs) linked with vancomycin. The traditional solid MBs have smooth surfaces and small specific surface areas, which limit the load capacity of the probes and make it difficult to achieve ultra-sensitive detection of large-volume targets such as Staphylococcus aureus. To overcome this drawback, this study adopted mesh-like MBs. Compared with solid MBs, their porous network provides a huge specific surface area, enabling high-density loading of metal-modified carbon dots and capture probes. This significantly improves the bacterial capture efficiency and the signal strength of the dual-modal detection. During the detection process, the MBs and CDs, as two detection probes, form a “sandwich sandwich” structure with the bacteria, and then are adsorbed by a magnet to separate from the supernatant. This platform shows excellent sensitivity for *S. aureus* and the dual-modal detection limit reaches 10 cfu/mL in the early warning and rapid detection of foodborne pathogenic bacteria, giving it broad prospects in this area.

## 2. Materials and Methods

### 2.1. Experimental Materials

The *S. aureus* (ATCC 65389) used in this experiment was provided by the Guangdong Institute of Microbiology (Guangzhou, China). Hydrogen peroxide (H_2_O_2_, 30%) was purchased from Sinopharm Chemical Reagent Co., Ltd. (Shanghai, China). 3,3′,5,5′-tetramethylbenzidine (TMB, 99%) and 1× PBS (pH = 7.6) were all purchased by Beijing Coolaber Technology Co., Ltd. (Beijing, China). N-Hydroxysuccinimide (NHS) and N-(3-dimethylaminopropyl)-ethyl carbodiimide hydrochloride (EDC) were purchased by Shanghai Aladdin Bio-chem Technology Co., Ltd. (Shanghai, China). Poly (glycolic acid) (PGA), HAc-NaAc buffer and Vancomycin (Van) were purchased from Shanghai yuanye Bio-Technology Co., Ltd. (Shanghai, China). Fe_3_O_4_ MBs were purchased from BaseLine Chromtech Research Centre (Tianjin, China); CuCl_2_ were purchased at Merck Reagents Co., Ltd. (Shanghai, China). *S. aureus* aptamer (Apt) (5′-COOH-GCAAT GGTAC GGTAC TTCCT CGGCA CGTTC TCAGT AGCGC TCGCT GGTCA TCCCA CAGCT ACGTC AAAAG TGCAC GCTAC TTTGC TAA-3′) was synthesized by Sangon Biotech Co., Ltd. (Shanghai, China). The Apt sequences used in this study have been previously reported and can specifically recognize Staphylococcus aureus. The binding affinity and specificity of these aptamers have been fully characterized [18]. All chemicals and solvents in this experiment were of reagent grade and used without further purification. Ultrapure water (18.2 MΩ) was used throughout the experiments.

### 2.2. Laboratory Instruments

Thermostatic culture shaker (BSD-YX2200, Shanghai Boxun Medical Biological Instrument Corp., Shanghai, China), CNC ultrasonic cleaner (KQ-300DA, Kunshan Ultrasonic Instrument Co., Ltd., Kunshan, China), metal bath (OSE-DB-01, Joan Lab Equipment (Zhejiang) Co., Ltd., Huzhou, China), electronic balance (UTP-313, Changzhou Xingyun Electronic Equipment Co., Ltd., Changzhou, China), biochemical incubator (SHP-80, Yangzhou Peiying Experimental Instrument Co., Ltd., Yangzhou City, China), ultra-clean bench (SW-CJ-1FD, Suzhou Purification Equipment Co., Ltd., Suzhou, China), high pressure steam sterilizer (YM50, Shanghai Sanshen Medical Equipment Co., Ltd., Shanghai, China), TU-1901 UV-vis spectrophotometer (PERSEE, Beijing, China), F97Pro fluorescence spectrometer (Lengguang technology Co., Shanghai, China), Zetasizer (Nano-Z, Malvern, UK), JEM-2100 transmission electron microscope (JEOL, Ltd., Tokyo, Japan), and freeze dryer (FD-1A-50, Shanghai Bilang Instrument Manufacturing Co., Ltd., Shanghai, China).

### 2.3. Synthesis of VAN/PGA@MBs

#### 2.3.1. Preparation of the Reticular MBs

Firstly, 200 μL of 10 mg/mL Fe_3_O_4_ MBs was taken and 900 μL of a mixture of 10 mM EDC and 5 mM NHS was added to activate the carboxyl groups of the MBs. The mixture was then shaken in a constant temperature shaker for 1 h. Then, 10 mg of PGA was added and the reaction was carried out in 1 mL PBS for 4 h, with the mixture constantly rotated. The PGA@Fe_3_O_4_ was synthesized. After the coupling reaction was completed, the unreacted PGA was washed away through magnetic force adsorption using PBS, and the reticular MBs were obtained.

#### 2.3.2. Preparation of VAN-Modified Macroporous MBs

The MBs were dissolved in 1 mL of PBS containing 2.90 mg of EDC and 3.26 mg of NHS to activate the carboxyl groups on PGA, and the mixture was incubated in a constant-temperature shaker for 2 h. The activated PGA@Fe_3_O_4_ precipitate was mixed with 0.9 mL of VAN solution (15 mg/mL) and incubated in a constant-temperature shaker for 4 h to synthesize VAN/PGA@MBs.

### 2.4. Synthesis of Cu@CDs/Apt

#### 2.4.1. Preparation of Cu@CDs

Next, 0.05 g citric acid, 0.07 g CuCl_2_, and 100.0 μL ethylenediamine were dissolved in 5.0 mL DL water. After uniform ultrasonic reaction, the reactants were transferred to the reactor, sealed, and placed in a 180.0 °C oven for 6 h, thus completing the preparation of Cu@CDs.

#### 2.4.2. Preparation of Cu@CDs/Apt

Apt was dissolved in 122 μL of water to obtain a 10 μM solution. The solution was heated in a metal bath at 95 °C for 5 min and then stored at 4 °C for 5 min. The Apt solution (122 μL) was mixed with 250 μL of EDC-NHS solution and incubated in a constant-temperature shaker for 30 min. Then, 500 μL of Cu@CDs was added to the mixture and incubated for 2.5 h in the shaker to synthesize Cu@CDs/Apt.

### 2.5. Condition Optimization

On the premise of determining the peroxidase-like (POD-like) activity of Cu@CDs, the quality of VAN/PGA/MBs and the concentration of Cu@CDs/Apt were optimized respectively to explore the optimal detection system of the colorimetric–fluorescence dual-modal sensor. Applying an external magnetic field causes the MBs to settle, thereby facilitating the collection of the supernatant liquid. The magnetic capture was carried out using the reverse magnetic adsorption method to obtain the supernatant. The magnetic capture time and incubation time of the nanoprobes were respectively optimized. To optimize the magnetic capture time, MBs were mixed with *S. aureus* and placed on a shaker. After magnetic adsorption for 10, 20, 30, or 40 min, unbound bacteria and MBs were removed by washing. The nano-signal probe was then added to the supernatant, and the mixture was incubated on a shaker for 30 min. The supernatant was collected for enzyme activity detection, and the signal was measured using a UV spectrophotometer. To optimize the incubation time, the magnetic capture time was fixed at 30 min. After washing, the nano-enzyme probe (Cu@CDs/Apt) was added and incubated for 10, 20, 30, or 40 min. The supernatant was then collected for enzyme activity detection, and the signal was measured using a UV spectrophotometer.

### 2.6. Construction of the Linear Regression Equation for S. aureus

Next, 100 μL of the targeted magnetic bead solution was added to 400 μL of different concentration bacterial solution (0, 10^1^, 10^2^, 10^3^, 10^4^, 10^5^, 10^6^, and 10^7^ cfu/mL). Then, 100 μL of Cu@CDs/Apt solution was added and incubated at 37 °C for 30 min. After magnetic separation, 600 μL of ultrapure water was added for resuspending and precipitation. After mixing evenly, 50 μL of the resuspended solution was added to 50 μL of H_2_O_2_ solution and 50 μL of TMB solution, and then 350 μL of HAc-NaAc buffer solution was added and incubated at 40 °C for 15 min. The ultraviolet absorbance value of the reaction solution at 653 nm was read using a UV spectrophotometer. Another 50 μL of the resuspended solution was added to 450 μL of PBS buffer solution and left to stand for 10 min. The fluorescence emission peak of different reaction solutions was read using a fluorescence spectrophotometer.

### 2.7. Specificity Assessment

The 10^4^ cfu/mL was operated according to the Section 2.6 with different pathogenic bacteria that have similar antigenic sites to *S. aureus*. The signal differences between the dual-modal sensor and *S. aureus* when detecting different foodborne pathogenic bacteria were read to evaluate the signal value of this sensing strategy.

### 2.8. Determination of Actual Samples

In this study, commercially available sausages were used as the experimental matrix. Firstly, the samples were initially tested using standard microbial culture methods to confirm that they did not contain the target pathogenic bacteria. Sausage samples were pretreated as described previously. In brief, 25 g of sample was minced and homogenized at high speed for 2 min. The homogenate was then mixed with 25 mL of PBS containing 3% (*w*/*v*) trichloroacetic acid and vortexed for 10 min to precipitate large molecules. After centrifugation at 4 °C, the supernatant was membrane-filtered and collected. Subsequently, an experimental model was established by adding artificially to confirm the detection and analysis of the samples.

## 3. Results

### 3.1. Characterization of VAN/PGA@MBs

**Figure 2:**
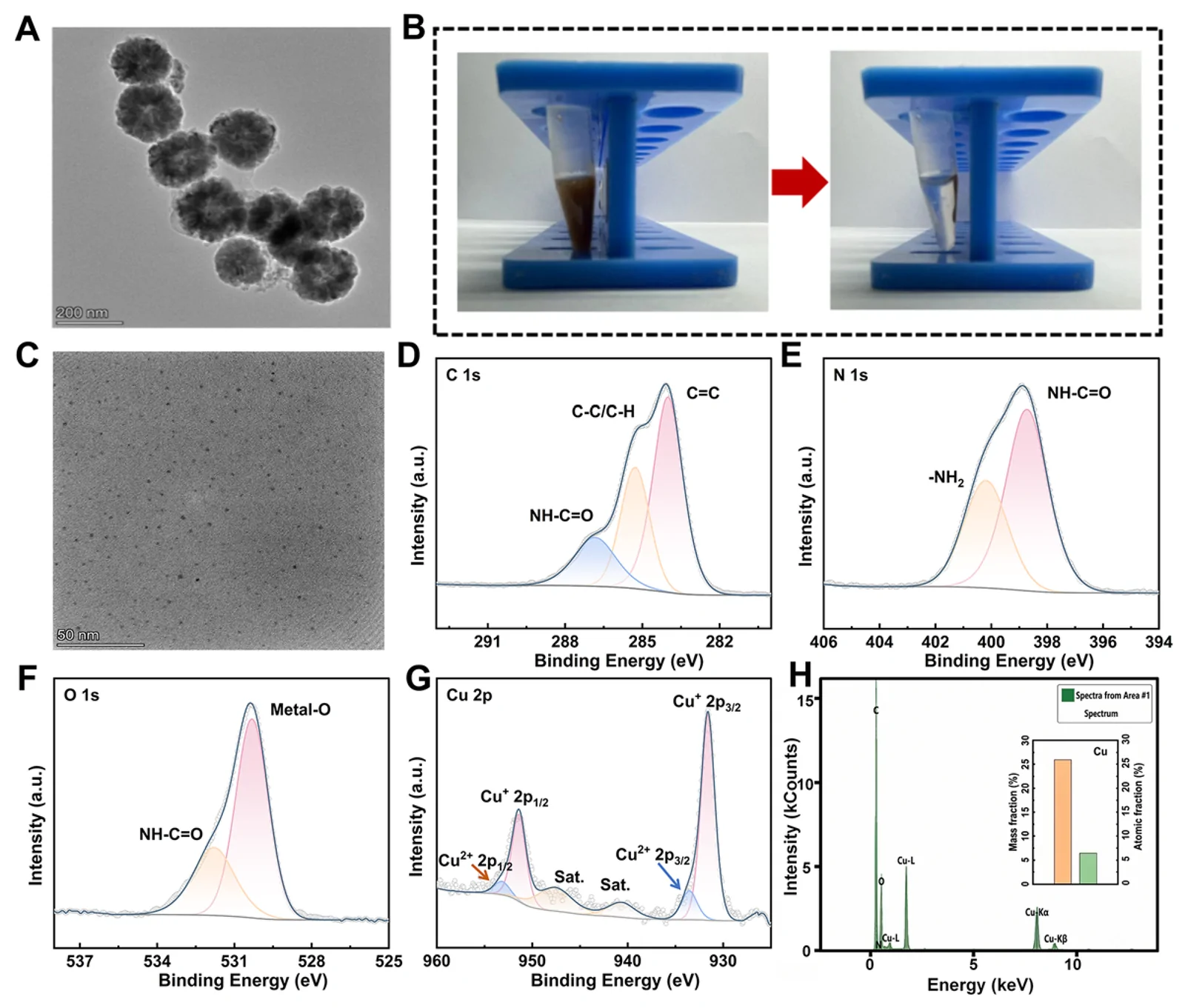
Characterization. (**A**) TEM image of VAN/PGA/MBs; (**B**) Magnetic analysis of VAN/PGA/MBs: magnetic bead dispersion (**left**) and aggregation (**right**) under an external magnetic field (blue frame, embedded magnet); (**C**) TEM image of Cu@CDs; (**D**–**G**) XPS spectra of Cu@CDs; (**H**) The elemental energy spectrum of Cu@CDs (on the left side of the illustration (orange), the representation indicates the mass fraction, while on the right side (green), it represents the atomic fraction).

The morphology and size of the VAN/PGA@MBs was characterized by scanning electron microscopy. The results are shown in Figure 2A. This complex is spherical in shape and has a distinct reticular structure. This is because the presence of PGA enables the formation of a stable reticular structure on the surface of the MBs [21], with an average diameter of approximately 200 nm. The adsorption and separation experiment further confirmed that the complex exhibits superparamagnetism and can be rapidly separated under an external magnetic field, enabling magnetic separation of the “sandwich structure” in subsequent detection (Figure 2B).

### 3.2. Investigation of the Properties of Cu@CDs

The characterization by high-resolution transmission electron microscopy (HRTEM) (Figure 2C) revealed that Cu@CDs are spherical nanoparticles with relatively uniform sizes, ranging from 2 nm to 3 nm in diameter. Subsequently, Cu@CDs were characterized by X-ray photoelectron spectroscopy (XPS). The C1s spectrum could be decomposed into three peaks, corresponding to C=C (284.00 eV), C-C/C-H (285.28 eV), and NH-C=O (286.83 eV) (Figure 2D). The N 1s spectrum showed two components at 398.72 eV and 400.19 eV, which were attributed to NH-C=O and -NH_2_, respectively (Figure 2E). In the O1s spectrum, the peak at 530.32 eV was attributed to the metal-oxygen bond (Metal-O), and the peak at 531.79 eV was attributed to NH-C=O (Figure 2F). The Cu 2p spectrum indicated that Cu mainly existed in the +1 oxidation state, with the relative contents of Cu^+^ 2p_3/2_ (931.61 eV) and Cu^+^ 2p_1/2_ (951.40 eV) totaling 86.49%; a small amount of Cu^2+^ was also present, with its 2p_3/2_ and 2p_1/2_ peaks located at 933.56 eV and 953.18 eV, respectively, and the total content was 13.51%. Additionally, the characteristic satellite peaks of Cu^2+^ were observed at 940.64 eV and 947.62 eV (Figure 2G). The EDS results further confirmed that the Cu element had been successfully doped into the CDs (Figure 2H). Cu doping endows carbon dots with the dual advantages of carbon-based and metal materials: the carbon skeleton provides excellent photostability, low toxicity and good biocompatibility, while the introduction of Cu species brings catalytic activity and electron transfer ability [22,23,24]. This synergy effect optimizes the material, further enhancing the catalytic efficiency of the nanomaterials and providing a crucial support for signal amplification.

#### 3.2.1. Exploration of Enzyme-like Activity of Cu@CDs

**Figure 3:**
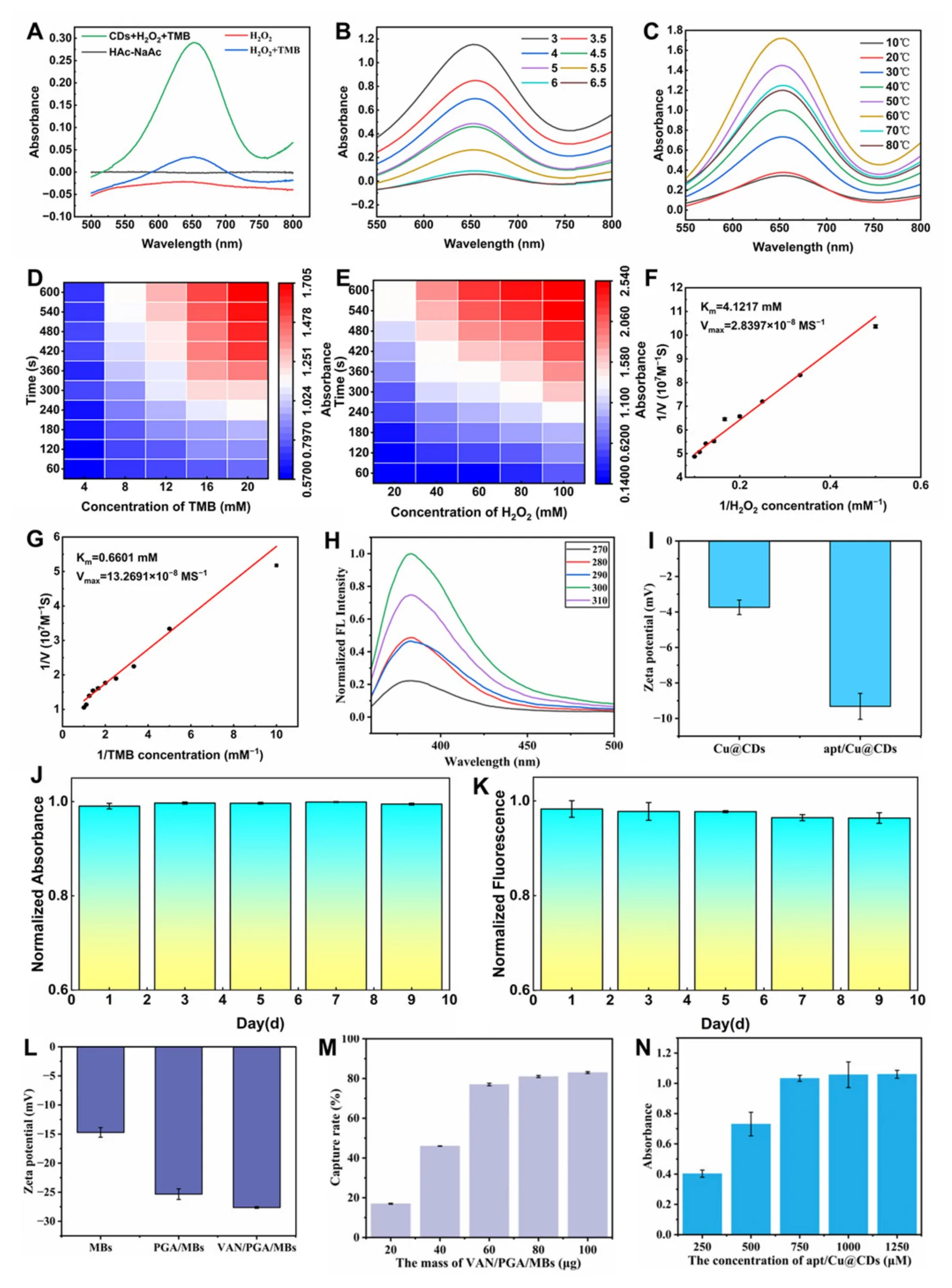
(**A**) Investigation of the enzyme-like activity of Cu@CDs under different conditions; (**B**) Optimization of pH of different buffers for Cu@CDs; (**C**) Optimization of the optimal catalytic temperature for Cu@CDs; Investigate the concentration dependence of TMB (**D**) and H_2_O_2_ (**E**) on the intensity of enzyme-like activity; Double inverted plots of plot of Cu@CDs@MPDA using H_2_O_2_ (**F**) and TMB (**G**) as the substrate; (**H**) Optimization of the optimal excitation wavelength; Zeta potential analysis of Cu@CDs/Apt (**I**) and VAN/PGA/MBs (**L**); Stability investigation of CDs: (**J**) enzyme-like activity, (**K**) fluorescence intensity; (**M**) Optimization of VAN/PGA/MBs quality; (**N**) Concentration optimization of Cu@CDs/Apt.

The enzyme-like activity of the synthesized nanomaterials was verified using an activity assay. A control group was prepared containing only 500 μL of pH 4.5 buffer solution. Three experimental groups were set up as follows: (1) 50 μL of H_2_O_2_ only; (2) 50 μL of H_2_O_2_ and 50 μL of TMB; (3) 50 μL of Cu@CDs solution, 50 μL of H_2_O_2_, and 50 μL of TMB. The final volume was adjusted to 500 μL with pH 4.5 buffer solution. After thorough mixing, the tubes were incubated in a metal bath at 60 °C for 15 min. The absorbance of the four groups was then measured (Figure 3A). The absorbance peak of the nanozyme at 653 nm wavelength was 0.29. The absorbance curve of the control group remained stable throughout the wavelength range and tended to the horizontal axis, which is in line with the theoretical expectation, proving that the nanozyme has POD-like activity. After confirming the enzyme-like activity of the nanozyme, we optimized the pH and temperature of the reaction system. Since the reaction solution system becomes black at pH 3 and pH 3.5, it may be because TMB is overoxidized or the nanozyme denatures under a more acidic pH. Therefore, the optimal pH for the POD-like activity of the nanozyme is 4 (Figure 3B). The temperature dependence of the nanozyme activity was evaluated across a gradient of 10, 20, 30, 40, 50, 60, 70, 80, and 90 °C. All other experimental conditions were kept constant, using a *S. aureus* concentration of 10^4^ CFU/mL. The optimal reaction temperature was determined by measuring the absorbance at 653 nm, and was found to be 60 °C (Figure 3C).

In order to systematically evaluate the POD-like catalytic activity of the synthesized nanomaterials, we quantitatively assessed their dose–response behavior towards H_2_O_2_ and TMB according to standardized detection conditions. Under the condition of a fixed nanomaterial dose and a constant incubation time (Figure 3D,E), as the H_2_O_2_ concentration increased from 20 mM to 100 mM, the absorbance showed a gradually increasing trend, which confirmed its strong concentration-dependent catalytic reaction. Similarly, when the H_2_O_2_ concentration reached the saturation value of 100 mM, the absorbance also increased within the TMB concentration range of 4 to 20 mM. In conclusion, these results indicate that these nanomaterials exhibit substrate concentration dependence. To further quantitatively evaluate the POD-like catalytic efficiency of the prepared nanomaterials, the steady-state kinetic parameters were determined in this study. Under optimal conditions, the concentration of the other substrate and the nanozyme dosage were kept constant while H_2_O_2_ or TMB was varied. The initial reaction velocities were recorded at different substrate concentrations. Linear fitting was performed using the Lineweaver-Burk double reciprocal plot, and the Michaelis constants (K_m_) and maximum reaction velocities (V_max_) of the nanomaterials for H_2_O_2_ and TMB were calculated respectively. The measured K_m_ value of the nanomaterials for H_2_O_2_ was 4.1217 mM, and for TMB was 0.6601 mM (Figure 3F,G). This result indicates that, compared with various recently reported nanozymes, the K_m_ values of Cu@CDs nanozyme for H_2_O_2_ and TMB were within an acceptable range, confirming its excellent affinity for the catalytic substrates and supporting the high sensitivity of this dual-mode sensing platform [25,26]. In addition, the stability of CDs was examined. During the 9-day test period, both the fluorescence intensity and the enzyme-like activity remained stable (Figure 3J,K).

#### 3.2.2. Investigation on Optimal Excitation

The fluorescence intensity was measured at excitation wavelengths of 270, 280, 290, 300, and 310 nm to determine the optimal excitation wavelength of the nanozyme. As shown in Figure 3H, the optimal excitation wavelength was determined to be 300 nm.

### 3.3. Synthesis and Characterization of VAN/PGA/MBs and Cu@CDs/Apt

The success of material synthesis was verified through changes in Zeta potential (Figure 3I,L). Firstly, after the MBs were modified with PGA, the surface negative potential significantly increased from −14.7 mV to −25.3 mV, proving the successful introduction of PGA. Subsequently, after VAN was loaded onto PGA, the potential of the VAN/PGA/MBs complex decreased from −25.3 mV to −27.6 mV, confirming the anchoring of VAN. Furthermore, the increase in the absolute value of the potential also indicates that the stability of the material has been enhanced compared to its original state [27]. Similarly, when the negatively charged nucleic acid Apt was fixed on the surface of Cu@CDs, the negative charge of Cu@CDs/Apt significantly increased, decreasing from −3.72 mV to −9.33 mV, indicating that the Apt had been successfully fixed.

### 3.4. Optimization of Detection Conditions

**Figure 4:**
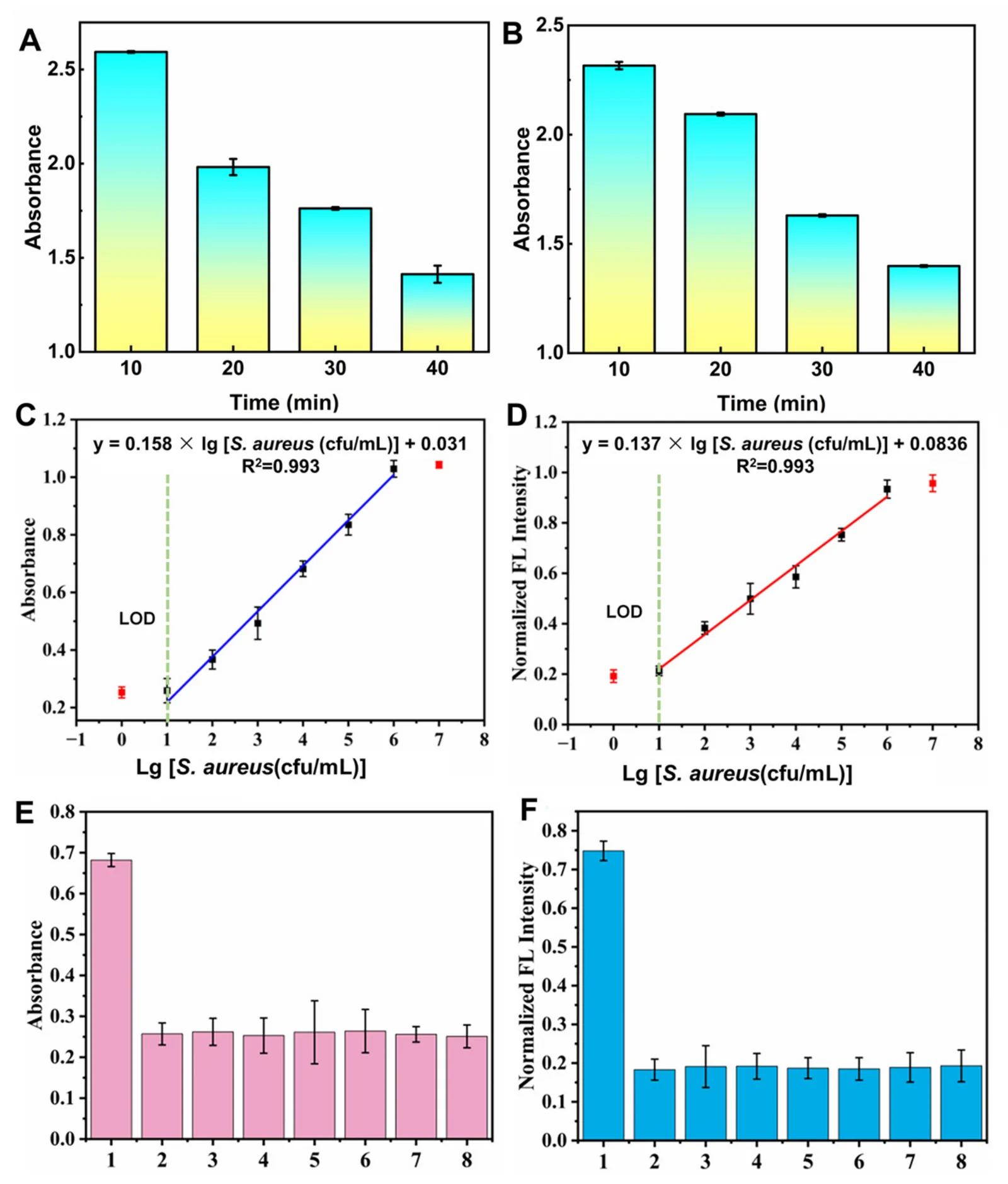
Sensitivity assessment and specificity analysis of the dual-modal sensor. (**A**) Optimization of incubation time; (**B**) Magnetic capture time optimization; (**C**) Linear equation of the colorimetric mode; (**D**) Linear equation of the fluorescence mode; (**E**) Colorimetric Mode Specificity Analysis; (**F**) Fluorescence Mode Specificity Analysis, 1–8: *S. aureus*, *Vibrio parahaemolyticus*, *Escherichia coli* O157:H7, *Succinic acid Clostridium*, *Pseudomonas aeruginosa*, *Enterococcus faecalis*, *Listeria monocytogenes*, and *Shigella flexneri*. Error bars represent the standard deviation of three independent measurements.

Under the optimal reaction conditions, the capture efficiency of VAN/PGA/MBs increased with the increase in their dosage, and it stabilized when the dosage reached 60 μg (Figure 3M). Based on these results and considering both the experimental efficiency and economy, this study ultimately selected 60 μg as the optimized quality of VAN/PGA/MBs. Furthermore, to determine the optimal concentration of Cu@CDs/Apt for screening, while keeping all other experimental conditions constant and the concentration of *S. aureus* (10^4^ cfu/mL) unchanged, five concentration gradients of 250, 500, 750, 1000, and 1250 μM were set for activity verification. The absorbance value at 653 nm (Figure 3N) was measured to determine that 750 μM was the optimal working concentration of Cu@CDs/Apt. To determine the optimal values for magnetic capture time and incubation time of the nanoprobes, we adopted a reverse magnetic attraction strategy for optimization. This strategy of collecting the supernatant was adopted to eliminate the optical background interference and strong light scattering caused by the dark-colored MBs, thereby maximizing the signal-to-noise ratio for the subsequent optical readouts. Based on the comprehensive experimental results, both were ultimately set to 30 min (Figure 4A,B).

### 3.5. Mechanism Investigation

If *S. aureus* is present in the detection system, the Cu@CDs/Apt will specifically capture *S. aureus*. After adsorption and separation with VAN/PGA@MBs, *S. aureus* can be enriched to reduce the interference of the meat matrix and improve the detection sensitivity [28,29]. Then, it can form a sandwich structure of Cu@CDs/Apt-*S. aureus*-VAN/PGA@MBs. In the sandwich detection structure constructed in this study, the nanozyme served as the core catalytic element and can efficiently catalyze the decomposition of H_2_O_2_ to produce highly active hydroxyl radicals. After adding the chromogenic substrate TMB, the redox reaction turned the solution light blue, providing a visual basis for colorimetric–fluorescence dual-signal detection [30]. In addition, the concentration of *S. aureus* in the detection system and the enrichment amount of nanozyme had a quantitative dependence at 653 nm, and this correlation can achieve quantitative detection of *S. aureus*.

### 3.6. Sensitivity Assessment of the Sensor

Under the optimal experimental conditions, to further evaluate the quantitative detection ability of the constructed nanomaterial biosensor, we investigated the relationship between the colorimetric–fluorescence intensity of the system and different concentrations of *S. aureus*. The absorbance of the reaction system was measured at 653 nm by UV-vis spectrophotometry, and the relationship between bacterial concentration and absorbance was established via a calibration curve. As shown in Figure 4C: the linear regression equation of the colorimetric mode was absorbance = 0.158 × lg [*S. aureus* (cfu/mL)] + 0.031, with limit of detection (LOD) of 10 cfu/mL. As the bacterial concentration increases, the fluorescence intensity of the system gradually enhances, and within a certain range of bacterial concentration, it showed a good concentration-dependent relationship. The linear regression equation of the fluorescence mode was fluorescence = 0.137 × lg [*S. aureus* (cfu/mL)] + 0.0836, with a LOD of 10 cfu/mL (Figure 4D). Therefore, the constructed nanoprobes can achieve sensitive detection of bacterial concentration through the enhanced response of colorimetry and fluorescence intensity.

### 3.7. Specificity Analysis

In order to verify the detection specificity of the constructed biosensor, several common foodborne pathogenic bacteria with similar antigenic epitopes to *S. aureus* were selected as potential interfering strains. The reaction tests were conducted under the same experimental conditions. As shown in Figure 4E,F, only in the presence of *S. aureus*, the system exhibited a significant absorbance and fluorescence enhancement response; while the signals changes caused by the other interfering bacteria strains showed no significant difference compared to the blank control. The above results indicate that this sensor can effectively identify the specific antigenic epitopes of the target bacteria, and does not show significant non-specific binding to other foodborne pathogenic bacteria with similar epitopes, demonstrating excellent detection specificity [18].

### 3.8. Actual Sample Assessment

*S. aureus* is a leading etiological agent of foodborne intoxication and healthcare-associated infections, underscoring its critical relevance in food safety, environmental monitoring, and clinical diagnostics [31,32]. However, real-world samples (e.g., raw milk, processed meat, environmental water, and human serum) contain complex matrices with abundant interferents, such as host proteins, lipids, polysaccharides, high ionic strength, and diverse non-target microbiota [33]. These matrix components can impede efficient bacterial capture, compromise signal generation fidelity, and ultimately degrade assay performance in terms of accuracy, LOD, and inter-sample reproducibility. Therefore, validation in authentic sample matrices is essential to assess the biosensor’s robustness against matrix interference and its practical applicability, and to facilitate its translation from proof-of-concept to real-world use. In this study, different concentration gradients of sausage samples were constructed and quantitative detection was carried out. As shown in Table 1, the colorimetry-fluorescence dual-modal sensor had a recovery rate of 95–105% in colorimetric mode and 95–100% in fluorescence mode, which fully verified that the detection system has a good quantitative ability. Its relative standard deviation (R.S.D.) ranges from 7.9% to 13.7%. Furthermore, in both the absorption and fluorescence signal modes, the relative standard deviation of the detection of Staphylococcus aureus by this detection mode remained at a relatively low level, indicating its excellent precision. We compared the detection limit, linear range, and actual sample spiked recovery rate of this method with similar detection methods in the recent literature (Table 2). The results showed that the performance indicators of this study were in a similar range to those of most existing reports, with the detection limit, linear range, and recovery rate all falling within the reasonable range reported in the literature.

## 4. Conclusions

In this study, the detection of *S. aureus* was accomplished using a dual-signal metal nanozyme probe through colorimetry and fluorescence dual signals. The developed dual-signal nanozyme probe showed a high degree of linear fitting, with a correlation coefficient of 0.993 obtained via linear regression in both colorimetric and fluorescence modes. This dual-modal sensor can achieve quantitative detection at 10 cfu/mL and had extremely high sensitivity. The dual-modal sensor provided two independent signal readings, which can be mutually referenced and compensated, significantly improving the accuracy and robustness of analysis in complex matrices. Compared with the time-consuming and labor-intensive traditional cultivation methods, the proposed bimodal sensing platform has significant advantages in terms of response time, cost-effectiveness and field portability. Given its excellent sensitivity and strong anti-interference ability, this smartphone-integrated device has broad application prospects in the rapid and immediate screening of Staphylococcus aureus in complex food matrices, effectively bridging the gap between high-end laboratory analysis and actual on-site monitoring.

## Figures and Tables

**Table 1:** Sample Analysis Table.

Sample	Add Label (cfu/mL)	Measured (cfu/mL)		Recovery (%, n = 3)		R.S.D. (%, n = 3)	
		Abs	Flu	Abs	Flu	Abs	Flu
Sausage	4.1 × 10^2^	4.3 × 10^2^	3.9 × 10^2^	105	95	13.7	12.8
	4.1 × 10^4^	4.2 × 10^4^	4.1 × 10^4^	102	100	11.2	9.7
	4.1 × 10^6^	3.8 × 10^6^	4.0 × 10^6^	95	98	11.2	7.9

**Table 2:** Comparison of the analytical performance of different nanomaterial-based methods for pathogenic bacteria detection.

Nano Materials	Detection Modal	Target Bacteria	LOD/LOQ (CFU/mL)	Detection Range (CFU/mL)	Matrix	Relative Standard Deviations (RSD)	References
Cu/Pt-graphene oxide (GO) nanosheets	Colorimetric	*Klebsiella pneumoniae*	20	20–2 × 10^7^	Clinical Samples	2.28%	[34]
	SERS		2	2–2 × 10^7^		1.19%	
Fe_3_O_4_@Zr-MOF/GOx	Colorimetric	*Salmonella typhimurium*	2.26	10^0^–10^6^	Raw milk	<9%	[13]
apt-PDA/AuPt	Colorimetric	*S. aureus*	44	1.4 × 10^2^–1.4 × 10^7^	Pork	1.93–7.66%	[35]
Apt@Sb@ZIF-90@hemin@ thioflavin T	Colorimetric	*Listeria monocytogenes*	10	10–10^6^	Milk and lettuce	3.2%	[36]
	Fluorescent					1.7%	
Tb-MOFs AuNPs	Fluorescent	*Pseudomonas aeruginosa*	0.63	1–10^6^	Bottled drinking water and orange juice	2.7–5.6%	[37]
GCDs-AgNCs	Fluorescent	*Listeria monocytogenes*	160	1.6 × 10^1^–1.6 × 10^6^	Lettuce and juice	-	[38]

## Data Availability

The data presented in this study are available on request from the corresponding author.

## Abbreviations

The following abbreviations are used in this manuscript:

*S. aureus*	*Staphylococcus aureus*
TMB	3,3′,5,5′-tetramethylbenzidine
CDs	Carbon dots
H_2_O_2_	Hydrogen peroxide
NHS	N-Hydroxysuccinimide
EDC	N-(3-dimethylaminopropyl)-ethyl carbodiimide hydrochloride
PGA	Poly(glycolic acid)
Van	Vancomycin
MBs	Magnetic beads
Apt	Aptamer
HRTEM	High-resolution transmission electron microscopy
XPS	X-ray photoelectron spectroscopy
LOD	Limit of detection
POD	Peroxidase
